# Three-Month Follow-Up of Heterologous vs. Homologous Third SARS-CoV-2 Vaccination in Kidney Transplant Recipients: Secondary Analysis of a Randomized Controlled Trial

**DOI:** 10.3389/fmed.2022.936126

**Published:** 2022-07-22

**Authors:** Andreas Heinzel, Eva Schrezenmeier, Florina Regele, Karin Hu, Lukas Raab, Michael Eder, Christof Aigner, Rhea Jabbour, Constantin Aschauer, Ana-Luisa Stefanski, Thomas Dörner, Klemens Budde, Roman Reindl-Schwaighofer, Rainer Oberbauer

**Affiliations:** ^1^Division of Nephrology and Dialysis, Department of Internal Medicine III, Medical University of Vienna, Vienna, Austria; ^2^Department of Nephrology and Medical Intensive Care, Charité-Universitätsmedizin Berlin, Freie Universität Berlin and Humboldt-Universität zu Berlin, Berlin, Germany; ^3^Department of Rheumatology and Clinical Immunology, Charité-Universitätsmedizin Berlin, Freie Universität Berlin and Humboldt-Universität zu Berlin, Berlin, Germany

**Keywords:** COVID-19, kidney transplantation, COVID-19 vaccination, heterologous vaccination, third vaccination

## Abstract

Response to SARS-CoV-2-vaccines in kidney-transplant recipients (KTR) is severely reduced. Heterologous3^rd^ vaccination combining mRNA and vector vaccines did not increase seroconversion at 4 weeks after vaccination, but evolution of antibody levels beyond the first month remains unknown. We have recently completed a randomized-controlled trial on heterologous (Ad26COVS1) vs. homologous (BNT162b2 or mRNA-1273) 3^rd^ vaccination in 201 KTR not developing SARS-CoV-2-spike-protein antibodies following two doses of mRNA vaccine (EurdraCT: 2021-002927-39). Here, we report seroconversion at the second follow-up at 3 months after the 3^rd^ vaccination (prespecified secondary endpoint). In addition, higher cut-off levels associated with neutralizing capacity and protective immunity were applied (i.e., > 15, > 100, > 141, and > 264 BAU/ml). A total of 169 patients were available for the 3-month follow-up. Overall, seroconversion at 3 months was similar between both groups (45 vs. 50% for mRNA and the vector group, respectively; *p* = 0.539). However, when applying higher cut-off levels, a significantly larger number of individuals in the vector group reached antibody levels > 141 and > 264 BAU/ml at the 3-month follow-up (141 BAU/ml: 4 vs. 15%, *p* = 0.009 and 264 BAU/ml: 1 vs. 10%, *p* = 0.018 for mRNA vs. the vector vaccine group, respectively). In line, antibody levels in seroconverted patients further increased from month 1 to month 3 in the vector group while remaining unchanged in the mRNA group (median increase: mRNA = 1.35 U/ml and vector = 27.6 U/ml, *p* = 0.004). Despite a similar overall seroconversion rate at 3 months following 3^rd^ vaccination in KTR, a heterologous 3rd booster vaccination with Ad26COVS1 resulted in significantly higher antibody levels in responders.

## Introduction

Vaccine response in kidney transplant recipients (KTR) is severely reduced due to the mandatory immunosuppressive medication following transplantation. Subsequently, a significant number of KTR remains at risk for SARS-CoV-2 infection despite vaccination (1, 2). Strategies to improve vaccine response in this high-risk population for severe COVID-19 are urgently needed.

We have recently conducted a randomized, single-blinded, controlled trial in 201 patients, comparing a homologous vs. heterologous vaccination strategy in KTR who did not develop SARS-CoV-2 spike protein-specific antibodies after two doses of an mRNA vaccine: Overall, 39% of patients developed antibodies at 4 weeks after the 3^rd^ dose, with no statistically significant difference between an additional dose of the same mRNA vaccine as used for the initial prime/boost vaccination (BNT162b2 or mRNA-1273, a 35% response rate) or a vector vaccine (Ad26COVS1, a 42% response rate) (3).

Other recent reports, however, have suggested a more pronounced induction of both, a SARS-CoV-2-specific CD4 T-cell response and antibodies, following heterologous vaccination that includes a vector-based vaccine in transplant recipients (4). In line, heterologous 3^rd^ vaccination also increased overall T-cell response in patients treated with B-cell-depleting therapy (5).

Most analyses to date were limited to observation within the first 4 weeks after 3^rd^ vaccination. Another recent observational study from France has reported changes in antibody levels in KTR from 1 month to 3 months after a 3^rd^ mRNA vaccine, showing a significant reduction in antibody levels (6). However, data on trajectories of antibody levels beyond the first month following heterologous vaccination remain unknown. In the current analysis of our randomized controlled trial (RCT), including follow-up data on antibody levels until month 3, we aimed to assess changes in antibody over time (month 1 to month 3) following homologous vs. heterologous 3^rd^vaccination. We hypothesized that a heterologous 3^rd^ vaccination using a vector vaccine would result in higher antibody levels at 3 months after vaccination compared to an additional homologous booster dose.

## Methods

### Study Cohort and Trial Design

Study participants were followed up for antibody assessment at the outpatient's transplant clinic of the Medical University of Vienna for a second follow-up (FU) between 60 and 120 days after the 3^rd^ vaccine dose (*3*-*month FU*, a pre-specified secondary endpoint). Details of randomization and treatment have been reported before (3). In short, 201 patients without detectable SARS-CoV-2 specific antibodies following two doses of a mRNA vaccine were randomized to a 3^rd^ dose of the same mRNA vaccine (the mRNA group) or a dose of the vector vaccine Ad26COVS1. Clinical endpoints (death, COVID-19) were recorded for all study participants throughout the observation period until 31^st^ of December 2021. The patients receiving a fourth vaccine dose or contracting COVID-19 before completion of the 3-month FU visit were excluded from analysis of vaccine efficacy.

### Assessment of the Humoral Response

Antibody response was evaluated using the Roche Elecsys anti–SARS-CoV-2 S enzyme immunoassay (Roche, Switzerland), detecting antibodies against the receptor-binding domain of the SARS-CoV-2 spike protein (the cutoff at 0.8 U/ml according to the manufacturer's instructions). As additional endpoints, we applied higher cut-off levels that were also reported as secondary endpoints at the 1-month FU and that are associated with neutralizing capacity as well as reduced risk for COVID-19 infection: > 100 U/ml (7), > 141 BAU/ml (8), and > 264 BAU/ml (9). BAU/ml were converted to U/ml based on the conversion formula: U/ml = 0.972^*^BAU/ml.

### Assessment of T-Cell Response

Besides the humoral response, we further analyzed SARS-CoV-2-specific CD4 and CD8 T-cell responses among humoral top responders at 4 weeks in both groups (*n* = 18 per group). The T-cell stimulation flow cytometric (FC) assessment of SARS-CoV-2-specific T-cells has been described before (10, 11). In brief, peripheral blood mononuclear cells (PBMC) were isolated by Ficoll-Paque density gradient centrifugation and cryopreserved until further analysis. For the identification of SARS-CoV-2-specific T-cells, 3–5 x 10^6^ PBMCs were incubated for 18 h with overlapping 15-mer peptides, covering the complete SARS-CoV-2 spike protein wild-type variant (1 ug/ml per peptide; JPT, Germany) and subsequently subjected to FC analysis. SARS-CoV-2-specific CD4 T-cells were identified based on CD154 and CD137 co-expression, whereas co-expression of CD137 and IFN-γ was used for CD8 T-cells. The gating strategy is exemplified in Supplementary Figure S1. The patients were considered having SARS-CoV-2-specific T-cells when the number of identified cells in the stimulated sample exceeded the number of such cells in the unstimulated sample by at least 2-fold. To account for patient-specific background activation, frequencies of activated cells detected in control samples were subtracted from the stimulated samples prior to any subsequent analysis of fractions of SARS-CoV-2-specific T-cells.

### Statistical Analysis

Patient demographics for continuous variables were reported as the median and interquartile range, except for patient age, which was reported as mean and standard deviation. Categorical variables were described by frequency and percentage. Differences between treatment groups for continuous and categorical variables were assessed by the Wilcoxon rank sum test and the Fisher's exact test, respectively. Occurrence of COVID-19 infections was visualized using a Kaplan–Meier graph. Wilcoxon rank sum tests were used for all comparison of absolute antibody concentrations as well as antibody level differences from 1-month to 3-month FU and detectable fractions of SARS-CoV-2-specific T-cells between groups. The number of seroconverted patients, number of patients with SARS-CoV-2-specific T-cells, and the number of patients exceeding defined antibody level cutoffs between groups were evaluated by means of the Fisher's exact test.

## Results

### Study Population

**Figure 1:**
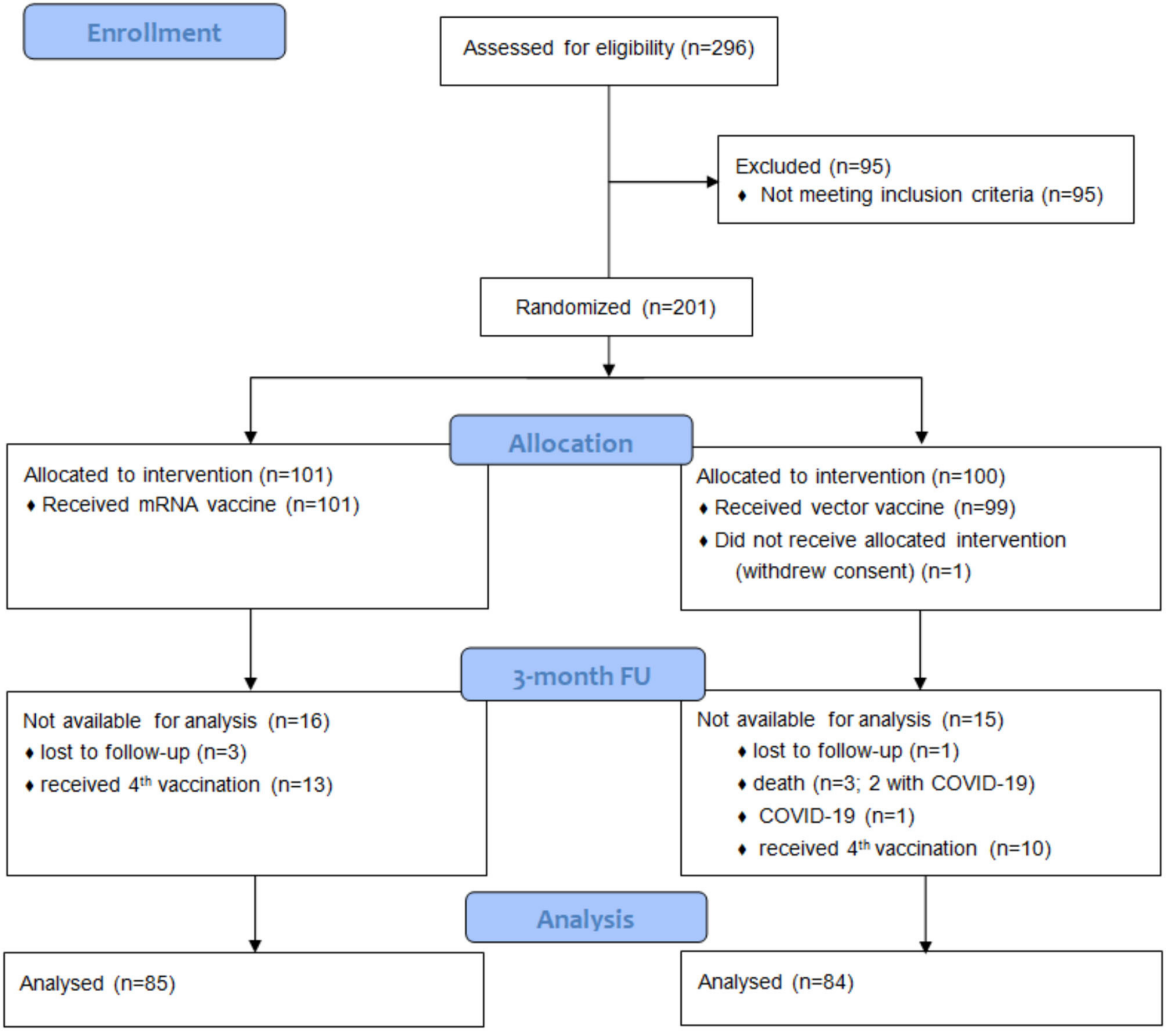
CONSORT flow chart for the 3-month follow-up. Blood samples for evaluation of vaccine efficacy at the 3-month FU were available for 169 of the initially enrolled 201 patients: One patient had withdrawn consent before vaccination, and 23 patients were excluded after they had received a 4^th^ vaccine dose before completing the 3-month FU visit; one patient died following myocardial infarction, two patients died due to COVID-19, one patient had mild COVID-19, and four patients had no blood draw within the observation period.

**Figure 2:**
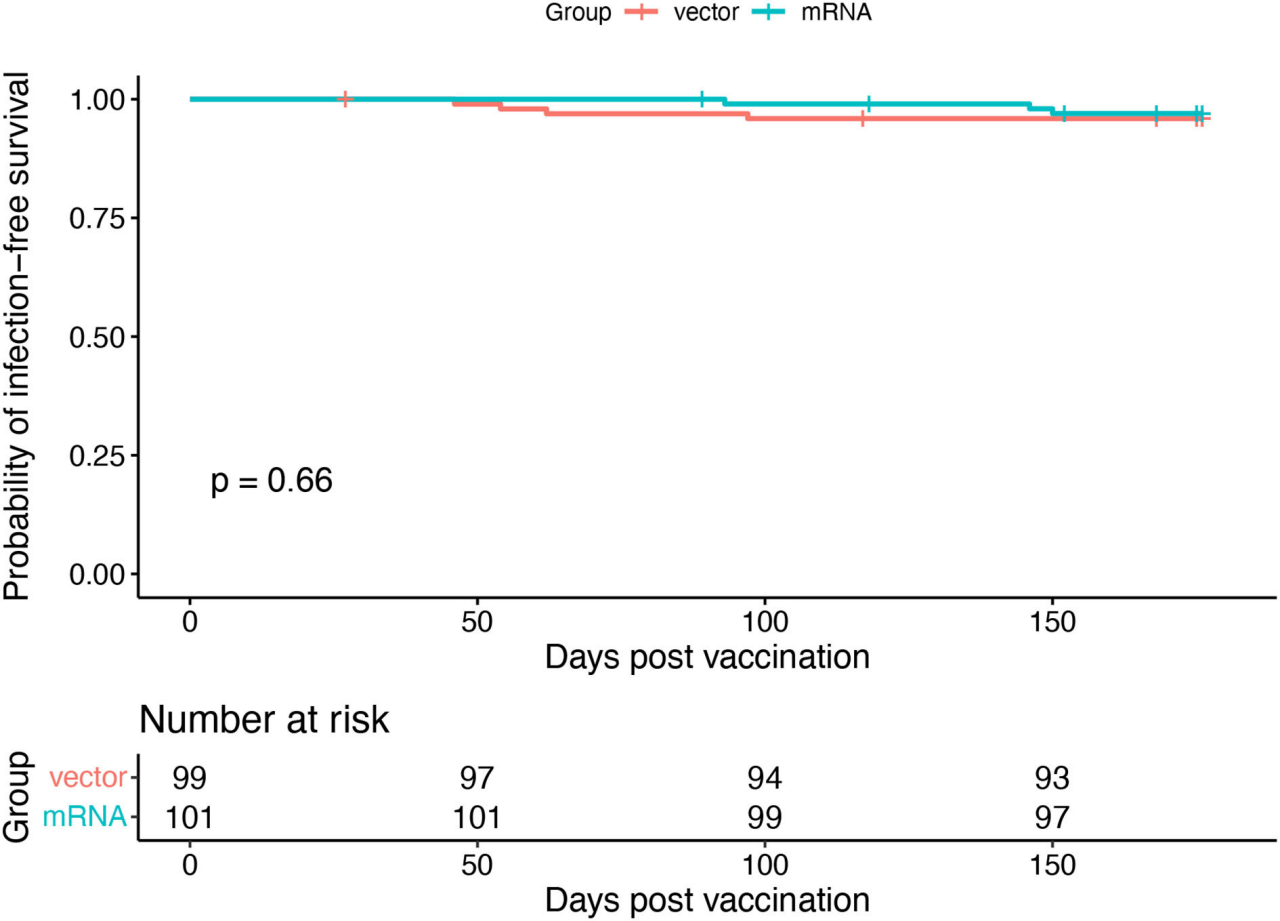
Occurrence of COVID-19 infections. A Kaplan–Meier graph of COVID-19 infection-free duration after homologous and heterologous third SARS-CoV-2 vaccination throughout the observation period. In case of non-COVID-19-related death, the follow-up period was censored at the date of death.

From the initially enrolled *n* = 201 patients, blood samples from 169 patients were available for the 3-month FU analysis of vaccine efficacy: 85 and 84 patients in the mRNA and vector groups, respectively (CONSORT Flow Chart is provided in Figure 1). Patient characteristics are provided in Table 1. There was no statistically significant difference between the mRNA and vector vaccine groups. Overall, eight deaths and seven SARS-CoV-2 infections occurred in the study population within the observation period (death: four vs. four; COVID-19: three vs. four for mRNA vs. vector vaccine groups, respectively; Figure 2). All COVID-19 cases occurred in vaccine no-/low-responders (six individuals without antibody response and one individual <15 U/ml); three patients had severe COVID-19, requiring ICU treatment (two patients in the vector group died as well as one patient from the mRNA group, who was on extra-corporal membrane oxygenation).

**Table 1:** Demographics of the study population.

**Variable**	**mRNA**	**Vector**
N	85	84
Mean (SD) age, y	61 (13)	61 (12)
Sex		
Female	37 (44)	34 (40)
Male	48 (56)	50 (60)
Time since KTX, y	4.8 [2.4–8.6]	4.9 [1.6–7.4]
No. of KTX		
1	64 (75)	66 (79)
2	15 (18)	13 (15)
3	4 (5)	4 (5)
4	2 (2)	0 (0)
5	0 (0)	1 (1)
Donor type (living)	14 (16)	18 (21)
Initial vaccinations (mRNA-1273)	27 (32)	27 (32)
Maintanance immunosuppression		
Belatacept, MMF, steroids	6 (7)	6 (7)
Belatacept, azathioprine, steroids	0 (0)	1 (1)
Cyclosporin A, MMF, steroids	1 (1)	4 (5)
Cyclosporin A, MMF	3 (4)	1 (1)
Cyclosporin A, azathioprine, steroids	1 (1)	0 (0)
MMF, steroids	1 (1)	1 (1)
Tracolimus, MMF, steroids	66 (78)	62 (74)
Tracolimus, MMF	1 (1)	3 (4)
Tracrolimus, azathioprine, steroids	4 (5)	3 (4)
Tracrolimus, steroids	2 (2)	2 (2)
Tracrolimus, leflunomide, steroids	0 (0)	1 (1)
ATG in past year	1 (1)	2 (2)
Nontriple immunosuppression	7 (8)	7 (8)
Time between second and third	78 [55–87]	80.5 [57–90.25]
vaccination, d
Time between third vaccination	31 [28–32]	30 [28–33]
and one-month follow-up visit, d
Time between third vaccination	81 [74–88]	76 [69–89]
and three-month follow-up visit, d

### Humoral Immune Response

The overall response rate to the 3^rd^ vaccine dose at the 3-month FU was 47%, with no statistically significant difference in seroconversion between the mRNA and vector vaccine groups [mRNA: 45% and vector: 50% OR = 1.24, 95% CI = (0.65, 2.37), *p* = 0.539]. Absolute antibody titers between the two groups were also not significantly different (median mRNA: 0.2 U/ml and vector: 0.81 U/ml, *p* = 0.104). However, when examining higher antibody cut-off levels that were also included in our primary analysis at the 1-month FU, we observed that a significantly higher number of patients in the vector group reached antibody levels above 141 and 264 BAU/ml [141 BAU/ml: 4 vs. 15% OR = 4.96, 95% CI = (1.29, 28.21), *p* = 0.009 and 264 BAU/ml: 1 vs. 10% OR = 8.75, 95% CI = (1.13, 396.17), *p* = 0.018, for mRNA vs. vector vaccine groups, respectively, Table 2]. In contrast, no difference between the groups was observed for any of the antibody level cut-offs at the 1-month FU (Table 2).

**Table 2:** The response rate to 3^rd^ SARS-CoV-2 vaccination at different pre-specified cut-off levels for the 1- and 3-month follow-up.

	**One-month FU**				**Three-month FU**			
**Cutoff**	**mRNA %**	**Vector %**	**P**	**OR 95%CI**	**mRNA %**	**Vector %**	**p**	**OR 95%CI**
0.8 U/mL	36	43	0.434	1.3 [0.67, 2.54]	45	50	0.539	1.24 [0.65, 2.37]
15 U/mL	22	26	0.594	1.23 [0.57, 2.66]	24	31	0.304	1.45 [0.7, 3.06]
100 U/mL	7	12	0.307	1.77 [0.55, 6.25]	8	17	0.108	2.22 [0.78, 6.89]
141 BAU/mL	5	8	0.37	1.83 [0.45, 8.89]	4	15	0.009	4.96 [1.29, 28.21]
264 BAU/mL	4	4	1	1.01 [0.13, 7.78]	1	10	0.018	8.75 [1.13, 396.17]

### Change in Serostatus Between Month 1 vs. Month 3

**Figure 3:**
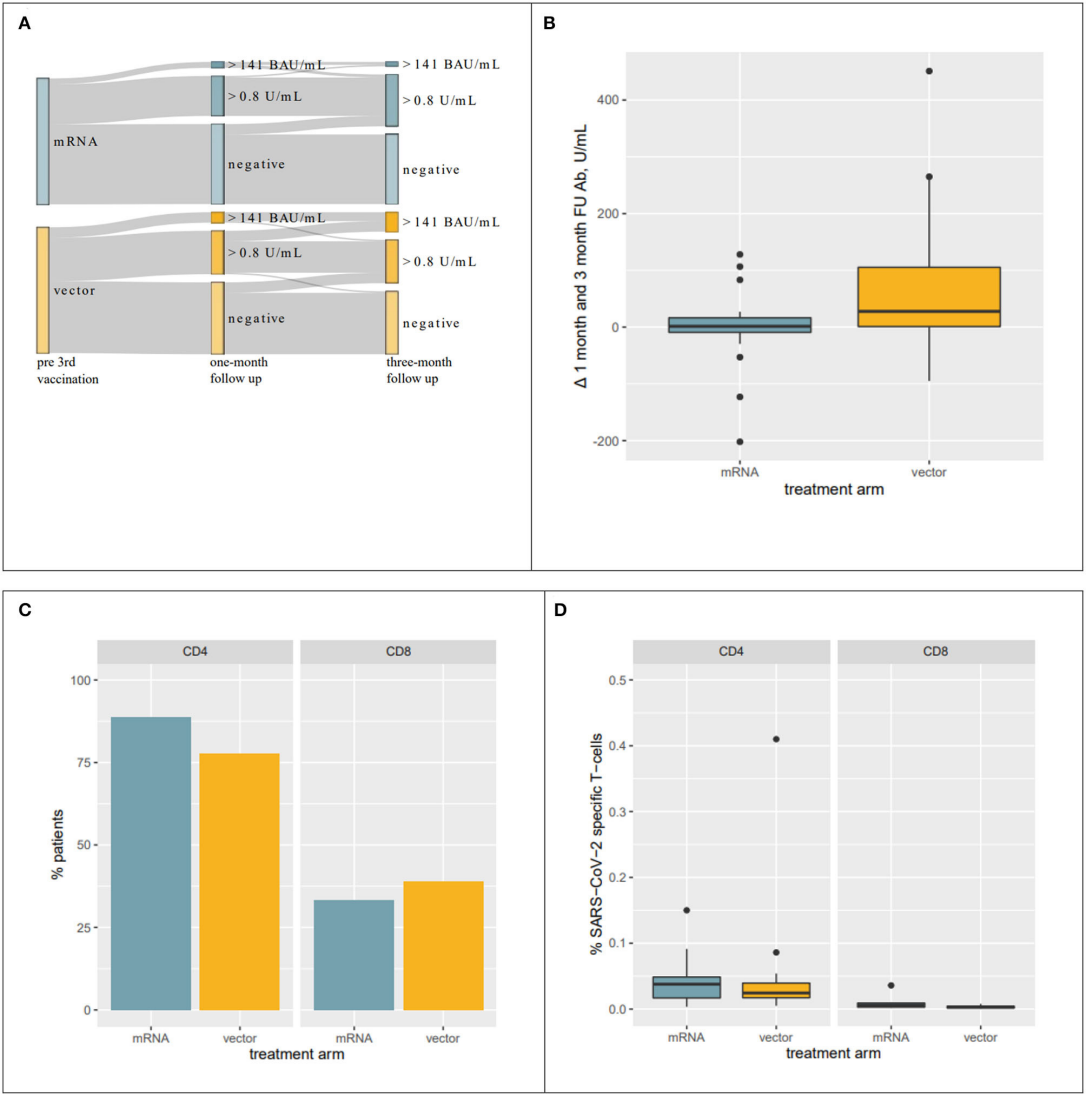
Response to vaccination. **(A)** Sankey Diagram visualizing changes in the response rate to 3^rd^ vaccination. A significantly larger proportion of individuals developed antibody levels > 141 BAU/ml. **(B)** Boxplots visualizing changes in antibody levels from 1- to 3-month FU in patients who seroconverted within 1 month after receiving their 3^rd^ vaccination. Antibody levels in individuals receiving a heterologous 3^rd^ vaccination further increased while remaining unaltered in patients receiving mRNA vaccines. **(C)** Percentage of patients with SARS-CoV-2-specific CD4 and CD8 T-cells among the top humoral responders at the 1-month FU. **(D)** Percentages of SARS-CoV-2-specific T-cells in patients with SARS-CoV-2-specific CD4 and CD8 T-cells.

In both groups, a comparable number of patients who had not seroconverted at the one-month FU became seropositive in the subsequent months [8 and 8% OR = 1.01, 95% CI = (0.29, 3.56), *p* = 1 for mRNA and vector, respectively]. With the exception of a single patient in the vector group, all the patients who showed seroconversion at the 1-month FU had antibody levels above the 0.8 U/ml cutoff at the 3-month FU. Figure 3A visualizes changes in serostatus, including increase above 141 BAU/ml as surrogate for protective immunity.

### Evolution of Antibody Levels Beyond the 1st Month

Of particular note, evolution of antibody levels in patients with seroconversion at the 1-month FU differed significantly between the two groups. Antibody levels in the vector group further increased after the 1-month FU while remaining approximately unchanged in the mRNA group (median of differences mRNA: 1.35 U/ml and vector: 27.6 U/ml, *p* = 0.004, Figure 3B). Consequently, absolute antibody levels were significantly different between the two treatment groups at the 3-month FU (median mRNA: 25.8 U/ml and vector: 77.7 U/ml, *p* = 0.038), even though they were not significantly different at the 1-month FU (mRNA: 19.7 U/ml and vector: 22.1 U/ml, *p* = 0.753).

### T-Cell Response

We also analyzed the T-cell response at the 1 month_FU in 18 patients among the top responders to the 3^rd^ vaccine from both groups to see if the subsequent increase in antibody levels in the vector group was preceded by a higher SARS-CoV-2-specific T-cell response. After the 3^rd^ vaccination, 83 and 36% of the patients had SARS-CoV-2-specific CD4 and CD8 cells, respectively. The number of patients with SARS-CoV-2-specific CD4 and CD8 T-cells was comparable between the treatment groups [CD4 mRNA: 89% and vector: 78% OR = 0.45, 95% CI = (0.04, 3.68), *p* = 0.658; CD8 mRNA: 33% and vector: 39% OR = 1.26, 95% CI = (0.27–6.19), *p* = 1, Figure 3C]. In the patients with SARS-CoV-2-specific CD4 and CD8 T-cells, a median of 0.033 and 0.003% overall CD4 and CD8 cells was SARS-CoV-2-specific. Interestingly, these numbers were also comparable between the two treatment groups (CD4 mRNA: 0.038% and vector: 0.024% *p* = 0.547; CD8 mRNA: 0.006% and vector: 0.003% *p* = 0.295, Figure 3D).

## Discussion

In this 3-month FU analysis of our RCT on homologous vs. heterologous 3^rd^ vaccination in KTR, we observed an increase in antibody levels from month 1 to month 3 in individuals receiving a heterologous 3^rd^ vaccination dose, with the vector vaccine Ad26COVS1. In contrast, antibody levels in individuals receiving a homologous 3^rd^ vaccination with an additional dose of mRNA remained unchanged from the 1-month FU to the 3-month FU, resulting in overall lower antibody levels in the homologous vaccination group. Consequently, there were a significantly higher number of individuals with antibody levels above antibody thresholds reported in the literature to be associated with neutralizing capacity despite a comparable overall seroconversion rate. Especially in the face of new variants that evade immune response (i.e., *Omicron* BA.1 and BA.2), higher antibody levels are needed for infection prevention, but cut-off levels conveying protective immunity remain undefined (12).

Interestingly, in both groups, 8% of KTR developed antibodies between completion of the primary endpoint at 4 weeks and the follow-up at 3 months. All the participants (excluding seven patients who tested positive for SARS-CoV-2 infection) had negative nucleocapsid antibody results at the 1- and 3-month follow-up, supporting delayed seroconversion rather than subclinical infections.

The difference in vaccine response > 141 BAU/ml and > 264 BAU/ml between both groups was only partly driven by an increase of responders in the heterologous vaccination group, but also a decline of antibody levels in individuals above these thresholds, following homologs vaccination. However, median antibody levels in the homologous vaccination group remained overall stable while increasing the vector group.

Overall, four percent of study participants contracted COVID-19 in the observation period. Clinical endpoints were similar between both intervention groups, and COVID-19 infections only occurred in no/low responders (<0.4 or <15 U/ml, respectively). Three KTRs had severe COVID-19, requiring intensive-care treatment, and two of these patients subsequently died. One fatality was in a vaccine low responder (5.9U/ml), suggesting that low-level antibody responses do not provide protection from severe disease. This is in line with reports that antibody levels > 141 or 264 BAU/ml are required for effective protection from symptomatic infections with the SARS-CoV-2 *alpha* variant (8, 9). We have previously compared antibody levels (BAU/ml) and neutralizing capacity in serum samples, following third vaccination: all samples with BAU > 141 BAU/ml also had neutralizing capacity (3).

Interestingly, there was no difference in the SARS-CoV-2-specific CD4 or CD8 T-cell response at 4 weeks after vaccination, comparing homologous or heterologous vaccination strategies. This contrasts with other reports in immunized individuals that suggest higher levels of T-cell response, following heterologous vaccination (5, 13), although clear thresholds or correlates of T-cell protection remain to be delineated. In animal models, adenovirus-based vector vaccines also induced a stronger T-cell response (14, 15). Data from the general populations show higher antibody and T-cell responses, following heterologous vaccination compared to homologous mRNA and vector vaccination strategies (16–18). However, most studies used the vector vaccine ChAdOx1 as opposed to Ad26COVS1. Overall, impact of heterologous vaccination on antibody levels in immunized patients was inconclusive, with some suggesting higher antibody levels in the heterologous group (KTR), while another showed a lower seroconversion rate in the heterologous vaccination group (patients treated with rituximab) (5, 13).

A limitation of this study is the incomplete follow-up as 23 patients had received a fourth vaccine dose before completing the 3-month FU and were, therefore, excluded from analysis of vaccine efficacy. However, the overall follow-up rate was still at 85% at 3-month FU. To identify a potential imbalance, we reanalyzed the primary endpoint at 1-month FU only including KTR who completed the 3-month FU and found in line with our previous report of the entire cohort no statistically significant differences between the treatment groups (Table 2). Applicability of previously identified antibody cut-off levels for infection prevention (i.e., > 141 BAU/ml or > 264 BAU/ml) to new immune-evasion variants (e.g., Omicron) remains unclear, and much higher levels are most likely required for protective immunity. Until now, no such cut-off levels have been reported in the literature. The primary objective of the present trial, however, was the comparison of the immune response, following homologous and heterologous vaccination: Increased immunogenicity of the heterologous vaccination approach may, therefore, also play an important role in the response to future variant-specific vaccines. In addition, different antibody detection platforms are used across the literature that shows different sensitivity or specificity to detect SARS-CoV-2 antibodies. All the samples were tested using the same platform, and we used the WHO standardized units reported as BAU/ml for the reported cut-off levels derived from the literature to allow for comparability across different platforms (19). The cut-off BAU <264 BAU/ml has been suggested as a cut-off to select individuals requiring additional immunization (20).

The strength of the study is the randomized controlled trial design and the pre-specified secondary endpoint at 3-month FU. To date, it has remained the only published RCT on heterologous third boost vaccination using Ad26COVS1 as a vector vaccine.

## Conclusion

Despite similar overall seroconversion rates and comparable antibody levels at 4 weeks, heterologous 3^rd^ boost vaccination using Ad26COVS1 results in significantly higher antibody levels in KTR over a 3-month follow-up period compared to additional homologous vaccination. More individuals in the heterologous vaccination group reached antibody levels associated with protective immunity against the SARS-CoV-2 *alpha* variant at 3 months.

## Data Availability Statement

The raw data supporting the conclusions of this article will be made available by the authors, without undue reservation.

## Ethics Statement

The studies involving human participants were reviewed and approved by Ethics Committee of the Medical University of Vienna. The patients/participants provided their written informed consent to participate in this study.

## Author Contributions

AH, ES, RO, and RR-S conceptualized the study and wrote the manuscript. FR, KH, LR, ME, CAi, RJ, CAs, A-LS, TD, and KB contributed to data acquisition, data analysis, and writing the of the manuscript. All authors contributed to the article and approved the submitted version.

## Funding

This study was supported by the Medical-Scientific Fund of the Mayor of the Federal Capital of Vienna (Project-Nr. 21182) and the Christine Vranitzky-Stiftung Research Grant 2020.

## Conflict of Interest

The authors declare that the research was conducted in the absence of any commercial or financial relationships that could be construed as a potential conflict of interest.

## Publisher's Note

All claims expressed in this article are solely those of the authors and do not necessarily represent those of their affiliated organizations, or those of the publisher, the editors and the reviewers. Any product that may be evaluated in this article, or claim that may be made by its manufacturer, is not guaranteed or endorsed by the publisher.
