# Corrigendum: Three-Month follow-up of heterologous vs. homologous third SARS-CoV-2 vaccination in kidney transplant recipients: Secondary analysis of a randomized controlled trial

**DOI:** 10.3389/fmed.2022.1048395

**Published:** 2022-10-10

**Authors:** Andreas Heinzel, Eva Schrezenmeier, Florina Regele, Karin Hu, Lukas Raab, Michael Eder, Christof Aigner, Rhea Jabbour, Constantin Aschauer, Ana-Luisa Stefanski, Thomas Dörner, Klemens Budde, Roman Reindl-Schwaighofer, Rainer Oberbauer

**Affiliations:** ^1^Division of Nephrology and Dialysis, Department of Internal Medicine III, Medical University of Vienna, Vienna, Austria; ^2^Department of Nephrology and Medical Intensive Care, Charité-Universitätsmedizin Berlin, Freie Universität Berlin and Humboldt-Universität zu Berlin, Berlin, Germany; ^3^Department of Rheumatology and Clinical Immunology, Charité-Universitätsmedizin Berlin, Freie Universität Berlin and Humboldt-Universität zu Berlin, Berlin, Germany

**Keywords:** COVID-19, kidney transplantation, COVID-19 vaccination, heterologous vaccination, third vaccination

In the published article, an author name was incorrectly written as “Schretzenmeier.”

The correct spelling is “Schrezenmeier.”

The authors apologize for this error and state that this does not change the scientific conclusions of the article in any way. The original article has been updated.

## Publisher's note

All claims expressed in this article are solely those of the authors and do not necessarily represent those of their affiliated organizations, or those of the publisher, the editors and the reviewers. Any product that may be evaluated in this article, or claim that may be made by its manufacturer, is not guaranteed or endorsed by the publisher.

