# Supplementary material for: Three-Month Follow-Up of Heterologous vs. Homologous Third SARS-CoV-2 Vaccination in Kidney Transplant Recipients: Secondary Analysis of a Randomized Controlled Trial
Source: Front Med (Lausanne). 2022 Jul 22;9:936126. doi: 10.3389/fmed.2022.936126 (PMC9353321; doi:10.3389/fmed.2022.936126)

Supplementary material to:

Three-Month Follow-Up of Heterologous vs. Homologous Third SARS-CoV-2 Vaccination in Kidney Transplant Recipients: Secondary Analysis of a Randomized Controlled Trial

Andreas Heinzel, Eva Schretzenmeier, Florina Regele, Karin Hu, Lukas Raab, Michael Eder, Christof Aigner, Rhea Jabbour, Constantin Aschauer, Ana-Luisa Stefanski, Thomas Dörner, Klemens Budde, Roman Reindl-Schwaighofer* and Rainer Oberbauer

Supplementary Figure S1:

FACS analysis scheme for identifying SARS-CoV-2 specific CD4 and CD8 T-cells based on co-expression of CD137 and CD154 and CD137 and IFN-γ, respectively.

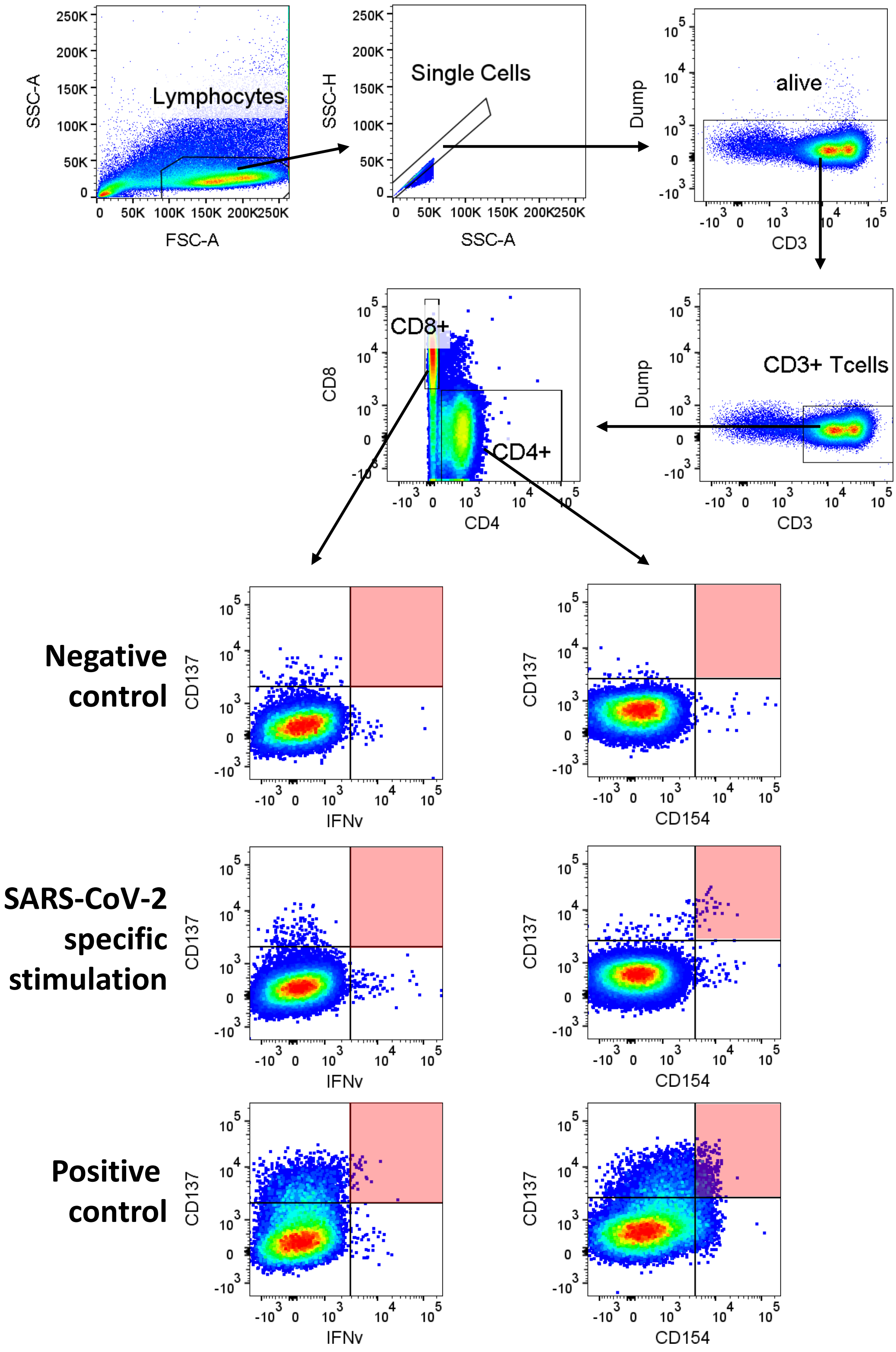

Supplement: Supplementary file 1 [file Image_1.pdf]
